# Intact Protein Analysis at 21 Tesla and X-Ray Crystallography Define Structural Differences in Single Amino Acid Variants of Human Mitochondrial Branched-Chain Amino Acid Aminotransferase 2 (BCAT2)

**DOI:** 10.1007/s13361-017-1705-0

**Published:** 2017-07-05

**Authors:** Lissa C. Anderson, Maria Håkansson, Björn Walse, Carol L. Nilsson

**Affiliations:** 1 Ion Cyclotron Resonance Program, National High Magnetic Field Laboratory, 1800 E. Paul Dirac Dr., Tallahassee, FL 32310 USA; 2 SARomics Biostructures AB, Medicon Village, SE-223 81 Lund, Sweden; 3 Department of Pharmacology and Toxicology, The University of Texas Medical Branch, 301 University Blvd., Galveston, TX 77555-1074 USA; 4 Institute of Clinical Sciences-Lund, Lund University, SE-221 85 Lund, Sweden

**Keywords:** Electron-transfer dissociation, Collisionally induced dissociation, High resolution, High field Fourier transform mass spectrometry, X Ray crystallography, Protein structure, Protein sequence variant, Precision medicine

## Abstract

Structural technologies are an essential component in the design of precision therapeutics. Precision medicine entails the development of therapeutics directed toward a designated target protein, with the goal to deliver the right drug to the right patient at the right time. In the field of oncology, protein structural variants are often associated with oncogenic potential. In a previous proteogenomic screen of patient-derived glioblastoma (GBM) tumor materials, we identified a sequence variant of human mitochondrial branched-chain amino acid aminotransferase 2 as a putative factor of resistance of GBM to standard-of-care-treatments. The enzyme generates glutamate, which is neurotoxic. To elucidate structural coordinates that may confer altered substrate binding or activity of the variant BCAT2 T186R, a ~45 kDa protein, we applied combined ETD and CID top-down mass spectrometry in a LC-FT-ICR MS at 21 T, and X-Ray crystallography in the study of both the variant and non-variant intact proteins. The combined ETD/CID fragmentation pattern allowed for not only extensive sequence coverage but also confident localization of the amino acid variant to its position in the sequence. The crystallographic experiments confirmed the hypothesis generated by in silico structural homology modeling, that the Lys59 side-chain of BCAT2 may repulse the Arg186 in the variant protein (PDB code: 5MPR), leading to destabilization of the protein dimer and altered enzyme kinetics. Taken together, the MS and novel 3D structural data give us reason to further pursue BCAT2 T186R as a precision drug target in GBM.

**Electronic supplementary material:**

The online version of this article (doi:10.1007/s13361-017-1705-0) contains supplementary material, which is available to authorized users.

**Graphical Abstract:**
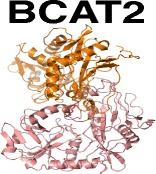
ᅟ

## Introduction

Structural technologies are an essential component in the design of precision therapeutics. Precision medicine entails the development of therapeutics directed toward a designated target protein, with the goal to deliver the right drug to the right patient at the right time. In the field of oncology, protein structural variants are often associated with oncogenic potential, such as BRAF V600E, a transduction protein variant targeted both by small molecule inhibitors and therapeutic antibodies [1, 2]. In the field of neuro-oncology, there is a dire need for new precision medicines to treat malignant primary brain tumors, especially glioblastoma (GBM), a tumor which carries a high risk of recurrence after standard of care therapy, and a nearly universal mortality within 5 y of diagnosis.

With a proteomic precision medicine strategy in mind, we identified the expression of approximately 225 single amino acid variants (SAVs) in a cohort of 36 brain tumor (GBM)-derived glioma stem cells (GSCs) by use of a proteogenomic approach [3]. Briefly, following validation by transcript matching (reads per kilobase of transcript per million mapped reads) to identified SAV peptides, we quantified the SAV peptides by multiple reaction monitoring. Whole exome sequencing was available for 20 of the patient samples, allowing us to differentiate between germline and somatic mutations. We validated and quantified variant proteoforms in the context of large sets of patient survival data contained in the Cancer Genome Atlas.

Our first identified potential target for GBM was branched-chain amino acid aminotransferase (BCAT) 2 T186R (UniProt, PDB: BCATM T159R). This germline protein variant was found in 25% of GSC lines, compared with an expected frequency of 9.6% in the population at large (1000 Genomes data [4]). Allelic imbalance, including loss of heterozygosity, was found in favor of the variant, and structural homology modeling of the variant protein implicated a significant change in the enzyme binding pocket [3]. BCAT2 is the human mitochondrial enzyme that degrades branched chain amino acids (Val, Ile, Leu) into branched chain α-keto acids (BCKAs) [5]. A second product of the reaction is glutamate, which is neurotoxic, allowing tumor expansion in the brain at the expense of normal brain cells. Thus, activity of BCAT2 may potentially fuel tumor growth through the further degradation of BCKAs and compromise normal brain tissue to favor tumor expansion. It is known that isozyme patterns of BCATs change in response to cell differentiation and carcinogenesis [5, 6]. BCAT1, the cytosolic form of the enzyme, has already been recognized as a therapeutic target of GBM [7]; however, that enzyme, although similar in its biochemistry, has little shared amino acid sequence with BCAT2.

The application of intact biomolecular dissociation in the gas phase by use of electron irradiation and heating techniques has been demonstrated to provide complementary datasets in the localization of protein features such as glycosylation [8, 9], phosphorylation [10, 11], and sulfation [12]. Electron transfer dissociation (ETD) [13–18] as well as ultraviolet photon dissociation (UVPD) [19, 20] have been applied successfully to top-down studies of proteins. The localization of single amino acid variants and potential post-translational modifications in intact proteins >30 kDa benefits greatly from top-down analysis in a high-field, high-sensitivity Fourier transform ion cyclotron resonance mass spectrometer. These MS methods provide confirmation of the amino acid sequence of the expressed proteins, which is synergistic to three-dimensional data and protein models derived from X-ray crystallography datasets.

To elucidate structural coordinates that may confer altered substrate binding or activity of the variant BCAT2 T186R, a ~45 kDa protein, we applied combined ETD, CID top-down mass spectrometry, and X-Ray crystallography in the study of both the variant and non-variant proteins. Top-down MS is an essential technology in the study of full length proteins, allowing for the detection of sequence variants, assignments of N- and C-termini, as well as post-translational modifications [21–27]. Sequencing of full-length gas-phase protein ions benefits from high field Fourier transform ion cyclotron resonance (FT-ICR) analysis, especially when coupled to a liquid chromatography system. We studied BCAT2 and BCAT2 T186R by LC-FT-ICR MS at 21 T equipped with multiple dissociation modalities (collisionally induced activation dissociation, CID; front-end electron transfer dissociation [28, 29] and a dynamically harmonized ICR cell [30–32]. At 21 T, the instrument routinely provides high mass resolution data on an LC timescale [32], even for large proteins [33, 34]. X-ray crystallographic coordinates are entirely complementary to MS data derived from full length proteins, providing data to construct three-dimensional images of protein structures.

## Experimental

### Protein Construct and Expression

**Figure 1:**
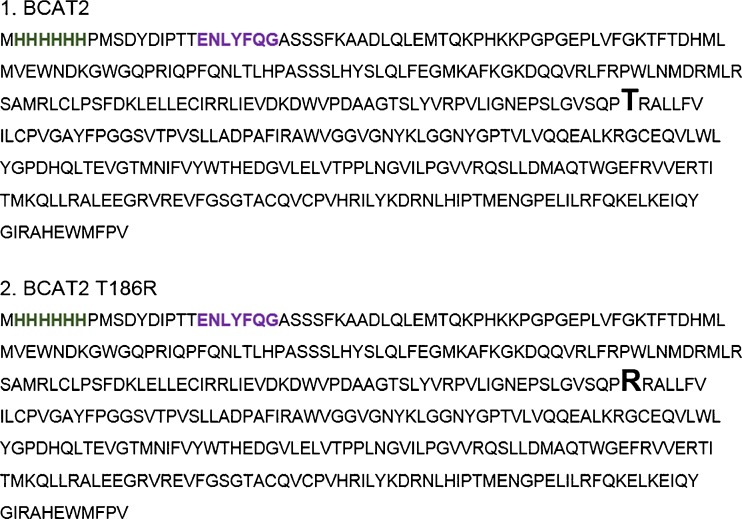
Sequences of expressed BCAT2 and BCAT2 T186R protein constructs. The location of the amino acid and variant (enlarged and in bold) is given as the number in the UniProt database (O15382). The His tag is in green and the TEV protease site in in purple bold text

Expression plasmids for production of human mitochondrial branched-chain aminotransferase (BCAT2) and a natural variant of it (T186R) were ordered from GenScript (oligonucleotide sequences are found in Appendix 1). BCAT2 proteins were fused to an N-terminal His-tag for affinity purification, followed by a tobacco etch virus (TEV) protease cleavage site for tag removal (Figure 1). Protein production was performed in Luria broth medium at 25 °C, 120 rpm in 5 L Erlenmeyer flasks. At OD600 = 2.0, isopropyl β-D-1-thiogalactopyranoside (IPTG) was added at a final concentration of 0.5 mM, and expression was allowed to continue for 20 h before the cells were harvested. After cell lysis and ultracentrifugation, BCAT2 proteins were purified on a 5 mL HisTrap HP column. Peak fractions were pooled and digested with TEV protease. After TEV digestion, the protein solution was passed a second time over the HisTrap column, and the flow-through and wash fractions were collected, pooled, and purified further on a gel filtration column. Finally, BCAT2 proteins were concentrated. We have used the UniProt numbering of the BCAT2 sequences (O15382) in this report.

### Liquid Chromatography

Immediately prior to LC-MS/MS, samples (0.5 μM) were incubated for 20 min at 55 °C in a solution containing 5 mM (Tris(2-carboxyethyl)phosphine), and 0.1% (v/v) acetic acid. Following incubation, these were acidified to pH 2 with formic acid. For each injection, 5 μL was loaded onto an in-house-fabricated 360 μm o.d. × 150 μm i.d. fused-silica micro-capillary trap column packed 3 cm with Poroshell 300-SB C8 resin (5 μm particle, 300 Å pore; Agilent Technologies, Palo Alto, CA, USA). The nano-HPLC system (ACQUITY M-Class; Waters, Milford, MA, USA) was operated at 2.5 μL/min for loading onto the trap column and washed with 95% A for 10 min. Separation was achieved in an in-house-fabricated 360 μm o.d. × 75 μm i.d. fused-silica micro-capillary analytical column packed 15 cm with C8 resin. Samples were eluted at 0.3 μL/min using a gradient of 5%–40% B in 5 min, 40%–70%B in 20 min, 70%–95% B in 5 min (30 min total length). The gradients utilized solvent A: 0.3% formic acid and 5% acetonitrile in water, and solvent B: 47.5% acetonitrile, 47.5% 2-propanol, 4.7% water, and 0.3% formic acid (% all expressed as v/v). Following separation, samples were directly ionized by micro-electrospray ionization using a 15 μm fused-silica PicoTip (New Objective, Woburn, MA, USA) emitter, which was packed with 2–5 mm PLRP-S resin (5 μm particle, 1000 Å pore, Agilent Technologies) to minimize the formation of bubbles and promote stable ESI.

### Mass Spectrometry

All data were acquired with a 21 T FT-ICR mass spectrometer described in detail elsewhere [32]. Briefly, the instrument design includes a dual cell, linear rf ion trap (Velos Pro; Thermo Fisher Scientific, San Jose, CA, USA) modified with a commercial Orbitrap Fusion API inlet and a front-end electron transfer dissociation (ETD) reagent ionization source [32]. A multipole storage device (MSD) is located behind the Velos Pro and is used to store multiple accumulations (“fills”) of analyte precursor or fragment ions prior to high-resolution mass analysis in the ICR cell [32, 35, 36]. This improves signal-to-noise more rapidly than spectral averaging and facilitates acquisition of tandem mass spectra (MS/MS) on a time scale that is chromatography-compatible.

Precursor (MS1) and product (MS2) ion spectra were collected in the ICR mass analyzer at 21 T. Instrument parameters were set as follows: for MS1 spectra – resolving power (RP) = 300,000 at *m/z* 400 (~762 ms transient duration), 1 × 10^6^ automatic gain control (AGC) target, 3 fills of the multipole storage device (3 × 10^6^ charges detected/transient), and 1–3 transients (micro-scans, μS) summed per spectrum. For MS2 spectra (isolation only or CID MS/MS) – RP = 300,000 at *m/z* 400, 7.5 × 10^5^ AGC target, 4–6 fragment ion fills of the MSD, 4 transients summed per spectrum, CID activation used 0% (isolation only) or 35% normalized collision, 10 ms activation period and 0.25 q, and 15 Th isolation window. The ETD experiments were conducted as described by Syka et al. [18], by use of an axial radio frequency (rf) pseudopotential for charge-sign independent trapping. The settings for ETD MS2 spectra – RP = 300,000 at 400 *m/z*, 4 E5 × 10^5^ reagent (fluoranthene) AGC target, 2 E5 × 10^5^ precursor AGC target, 15 fragment ion fills of the multipole storage device, 4 transients summed per spectrum, and 5 ms ETD reaction time.

### Mass Spectrometric Data Analysis

Data were manually interpreted by use of Xcalibur 2.1 software (Thermo Fisher Scientific). Accurate masses for intact BCAT2 proteins were calculated with IsoPro ver. 3.1 (Senko, M.W., https://sites.google.com/site/isoproms/). Xtract (Thermo Fisher Scientific) parameters were set as follows: M (neutral) masses generated from deconvolution, fit factor 44%, remainder 25%, resolution at *m/z* 400 set to 300,000, S/N threshold 3, and max charge 60.

### Crystallization and X-ray Data collection

Purified BCAT2 protein (11 mg/mL) and BCAT2 T186R protein (9.3 mg/mL) in buffer (25 mM HEPES pH 7.5, 25 mM NaCl, 20 mM EDTA, 2.5% glycerol, 20 mM DTT) were used for crystallization experiments. The crystals were grown at 20 °C from the pH, anion, cation crystallization trial screen, reservoir (0.2 M NaF, 0.1 M Bis Tris propane buffer, pH 6.5, and 20% w/v PEG 3350) supplemented with 10 mM dithiothreitol, as performed in previous successful crystallization experiments of BCAT2 non-variant protein [37]. The crystals appeared within a few days and grew to 0.1 × 0.1 × 0.1 mm. A crystal was harvested by addition of reservoir mixed with ethylene glycol (final concentration of 24% v/v ethylene glycol) and flash-frozen in liquid nitrogen. Data were collected at 100 K at Diamond Light Source (UK), beamline i03. Data were processed in XDS [38] and Aimless [39] to 1.6 Å. The structure was determined with Molecular Replacement in Phaser [40] and the human BCAT2 structure as a model with pdb code (5CR5). The link between pyridoxal phosphate (PLP) and K202 (Uniprot K229) was created using the Jligand software [41]. The structure was refined in Refmac [42] and model building was carried out in Coot [43]. The statistics for the data collection and refined coordinates are given in Supplementary Table 1 (Supporting Information). The structure has been checked by use of the Molprobity server [44]; 97.9% of all amino acids are in the preferred regions of the Ramachandran plot and the rest are in allowed regions. The BCAT2 T186R structure has been deposited in the protein data bank with pdb code (5MPR).

## Results

### Mass Spectrometric Analysis

**Figure 2:**
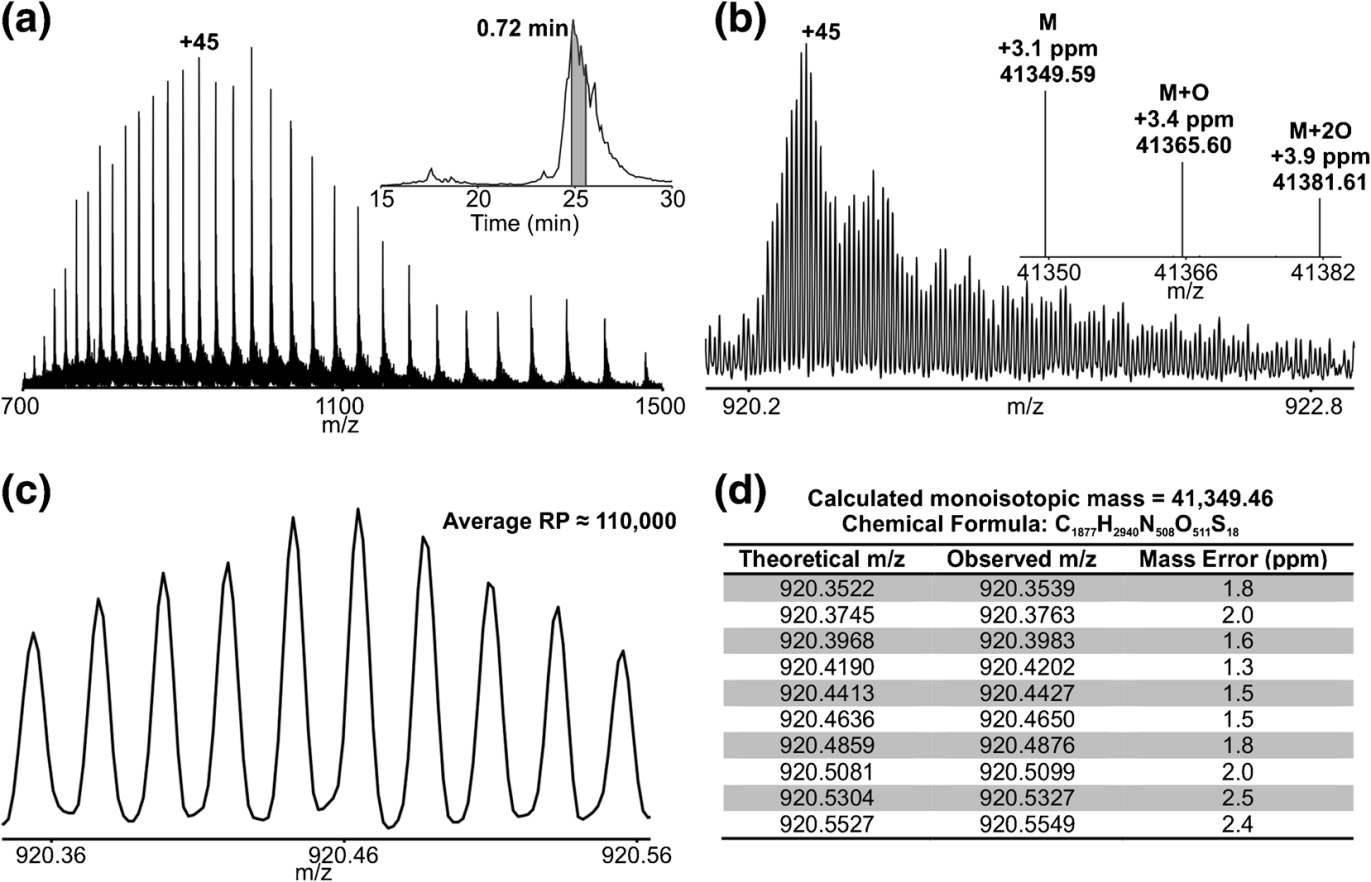
(**a**) Broadband high resolution mass spectrum of BCAT2 T186R using 0.76 s transients (three microscans/spectrum; post-FT average of 10 spectra). Inset shows region of chromatogram over which data shown was acquired. (**b**) Expanded view of the +45 charge indicated in (**a**). Inset shows the three most abundant species observed in the Xtract deconvolved spectrum derived from (**a**). (**c**) Expanded view showing the 10 most abundant isotopologues of BCAT2 T186R. (**d**) Table of theoretical and observed *m/z* for the 10 isotopologues displayed in (**c**) as well as mass error (ppm). The RMS error for the 10 peaks shown was 1. 9 ppm

The mass analysis of the TEV-cleaved BCAT2 variant protein by LC-MS in the 21 T FT-ICR MS system (Figure 2) yielded high resolution (resolving power 110,000 at *m/z* 920) and high mass accuracy molecular weight data (monoisotopic mass 41,349.46 Da). The analysis was performed on a true-LC timescale; the spectrum is the average of just 10 MS1 scans (*m/z* 700–2000; 30 total transient acquisitions), taking less than 45 s to acquire. Note that mass errors depend on whether averagine fits are used to determine monoisotopic mass. For example, manual examination of the 10 most abundant isotopic peaks (Figure 2c) of the [M + 45H]^45+^ prior to deconvolution yields 1.9 ppm RMS mass error using the known elemental composition (Figure 2d), whereas monoisotopic mass assignment by Xtract deconvolution leads to 3.1 ppm mass error (Figure 2b). Better mass accuracy is achieved by final comparison to the assigned protein elemental composition, rather than the averagine elemental formula [34].

**Figure 3:**
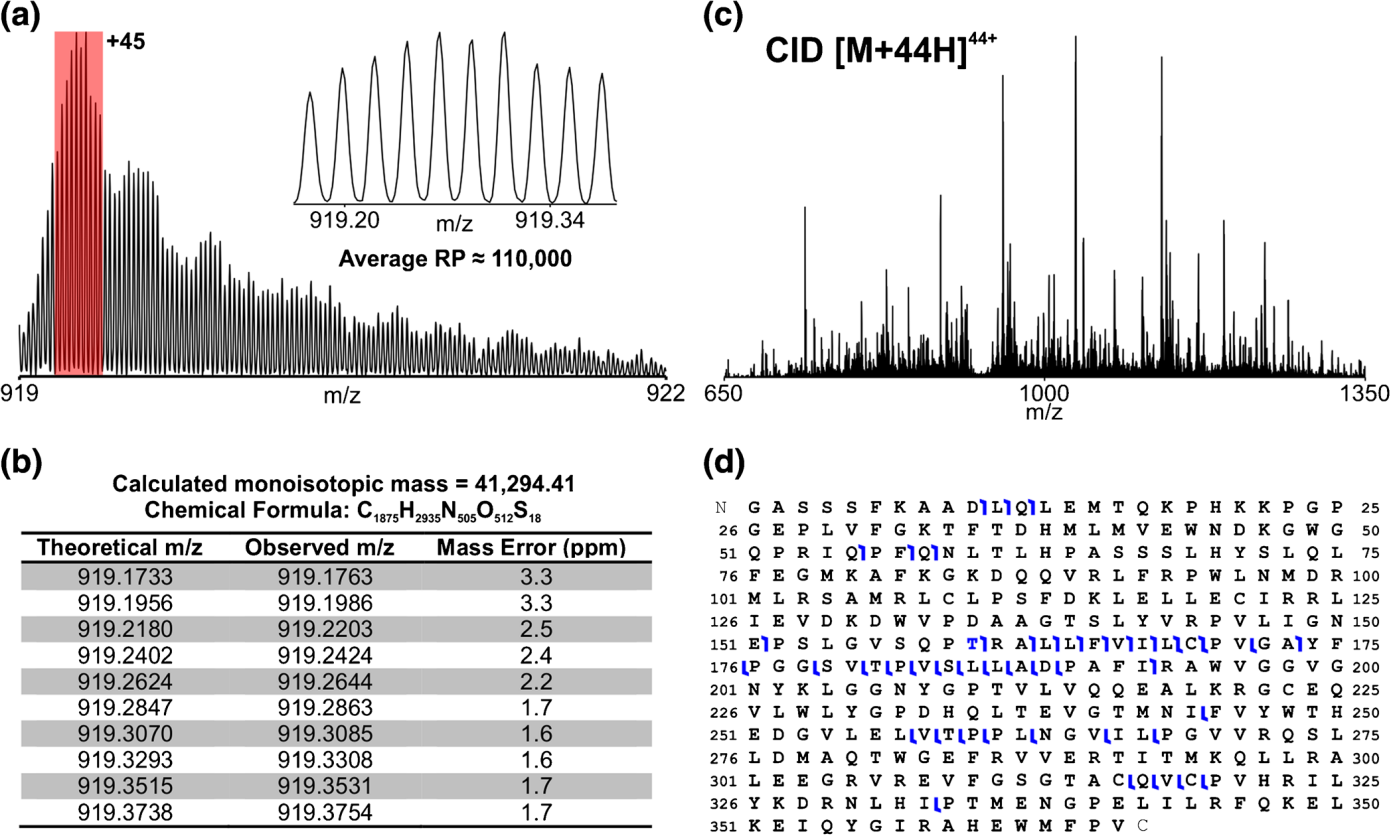
(a) Mass spectrum of the isolated BCAT2 WT [M + 45H]^45+^ acquired with four 0.76 s transients. Total data acquisition time was approximately 5.4 s (four fills of multipole storage device; 7.5 × 10^5^ precursor AGC target; 3 × 10^6^ charges detected). Inset shows expanded view of the 10 most abundant isotopologues of the distribution (shaded red). (**b**) Table of theoretical and observed *m/z* for the 10 isotopologues displayed in (**a**) as well as mass error (ppm). RMS error for the 10 peaks shown was 2.3 ppm. (**c**) CID MS/MS spectrum of BCAT2 WT [M + 45H]^44+^ precursor using 0.76 s transients (four microscans/spectrum; post-FT average of three spectra). Total data acquisition time was approximately 16 s (~5.4 s/spectrum; four fragment ion fills; 7.5 × 10^5^ precursor AGC target). (**d**) Sequence coverage derived from the spectrum and the position of the single amino acid substitution are indicated

Equivalently resolved isotopic distributions with comparable mass accuracies to those observed in Figure 2 are obtained upon 15 Th isolation of the [M + 45H]^45+^ charge state of the non-variant protein by summing just 4 transients (5.4 s total acquisition time; Figure 3a and b). This demonstrates the utility of 21 T FT-ICR for rapid determination of intact protein mass on an LC timescale. The observed mass deviation between the two BCAT2 proteoforms corresponded to a threonine-to-arginine substitution in the protein sequence. However, it is not sufficient to fully characterize variant protein sequences based on intact mass measurements alone. For this task, MS/MS analysis of the protein is required. This is typically performed with a bottom-up approach, where protein disulfide bonds are reduced, alkylated, and the protein digested into peptides by use of trypsin. The peptides are then analyzed by LC-MS/MS. This is a time-consuming, labor-intensive process. Because intact protein mass must be measured anyway, a simpler approach, from a sample preparative standpoint, is to just fragment the intact protein. Because the protein mass is greater than 30 kDa, we were strongly motivated to perform the MS/MS analysis in the 21 T instrument, and to combine two methods of dissociation in order to maximize sequence coverage with the goal of demonstrating the rapid speed with which the desired data can be acquired.

**Figure 4:**
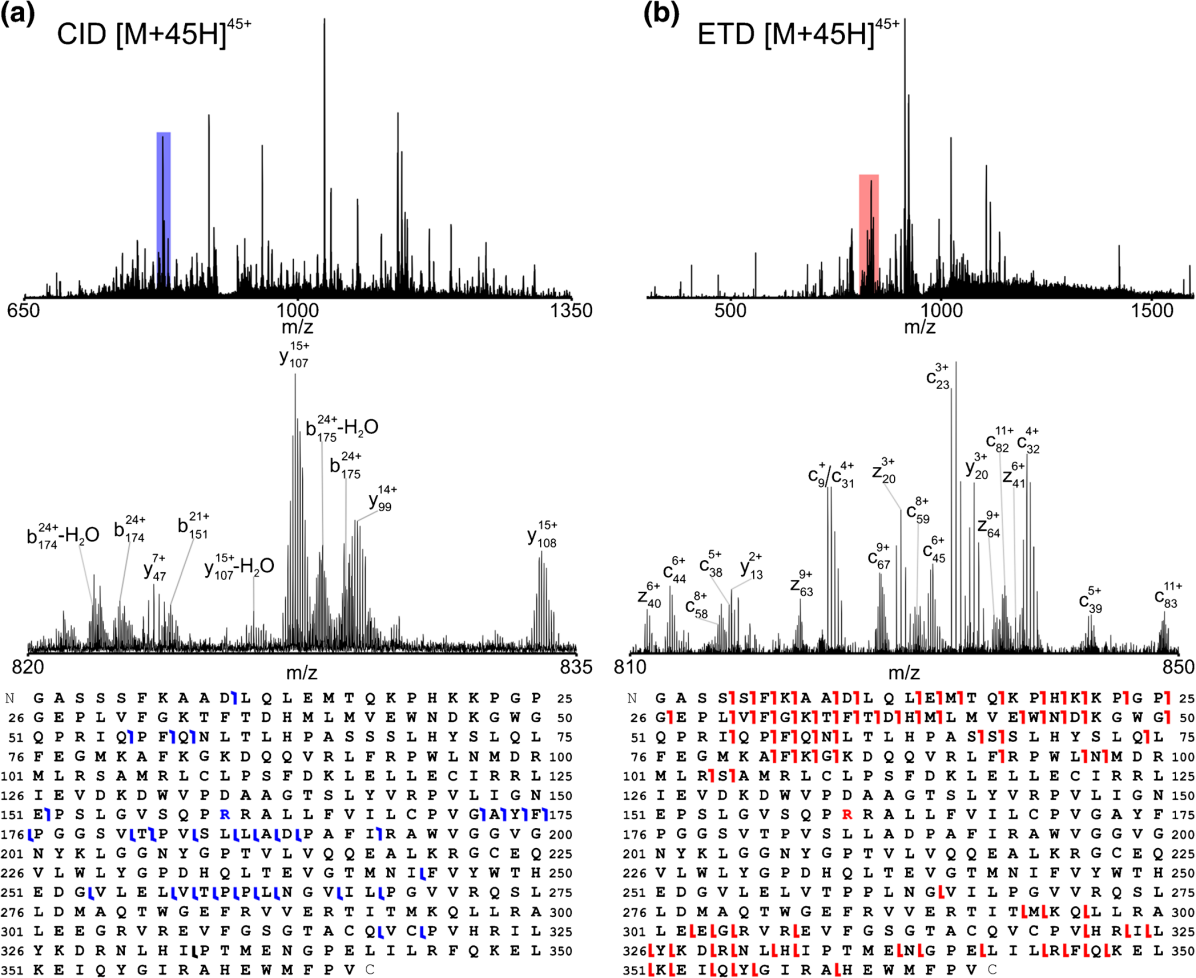
(**a**) (top) CID MS/MS spectrum of BCAT2 T186R [M + 45H]^45+^ precursor using 0.76 s transients (four microscans/spectrum; post-FT average of three spectra). Total data acquisition time was approximately 21 s (~7 s/spectrum; six fragment ion fills; 7.5 × 10^5^ precursor automatic gain control (AGC) target). (**b**) (Top) 5 ms ETD MS/MS spectrum of BCAT2 T186R [M + 45H]^45+^ precursor using 0.76 s transients (four microscans/spectrum; post-FT average of three spectra). Total data acquisition time was approximately 42 s (~14 s/spectrum; 15 fragment ion fills; 2 × 10^5^ precursor AGC target). Zoom insets of the regions shaded in color are shown center with fragment ions labeled. Sequence coverage derived from each spectrum and the position of the single amino acid substitution are indicated (bottom)

We performed CID and ETD experiments on the 45+ charge state of the variant protein (Figure 4a and b, respectively). Again, limited spectral averaging was utilized to demonstrate chromatographic compatibility; the spectra are the post-FT spectral average of just three scans (4 transients summed/scan) and took a combined total of 21 s (panel a) and 42 (panel b) to acquire. This difference in spectral acquisition speed is attributable to the difference in appropriate precursor ion populations for CID versus ETD fragmentation as performed on this instrument. ETD requires that precursor ions be sequestered in the rear section of the linear ion trap so that reagent ion injection and isolation can occur in the center section [18]. The rear section exhibits a diminished capacity to store ions and so smaller precursor ion populations are used and signal-to-noise ratios of the resulting fragment ion signals suffer. Thus, more fragment ion fills (15 fills for ETD versus 6 fills for CID) of the MSD are utilized to compensate, resulting in longer spectral acquisition times. Despite this increase, ETD MS/MS spectra are acquired rapidly enough for on-line analyses (as demonstrated here). CID and ETD MS/MS fragments were manually assigned to the protein sequence from the unprocessed spectra. Table 1 lists example ETD fragments through charge state 3^+^ for which the monoisotopic peak was observed and gives calculated ppm errors of fragments using their respective elemental compositions (monoisotopic peak error) and as determined by Xtract deconvolution (RMS errors 1.2 and 1.8 ppm, respectively). Sub-ppm mass accuracy was observed in the unprocessed spectrum for 20 of the 28 fragments shown. Note that in addition to the improved mass accuracy, several fragment ions are not observed upon deconvolution, presumably because these fell below the default S/N threshold or they were not properly deconvolved.

**Table 1:** Examples of BCAT 2 T186R ETD Fragment Mass Errors from Monoisotopic Peaks (Prior to Deconvolution) and Xtract Deconvolution

Ion type	Observed *m/z*	Monoisotopic peak error (ppm)	Xtract deconvolution error (ppm)
C4	320.1567	0.6	–
C5	407.1887	0.5	–
C6	554.2572	0.5	–
C7	682.3521	0.3	–
C9	824.4267	0.7	1.0
C10 2+	470.2304	0.4	0.8
C13	1293.6826	2.2	2.4
C14	1422.2772	3.4	3.6
C14 2+	711.8652	0.6	
C15 2+	777.3855	0.6	–
C17 2+	891.9388	0.7	0.9
C19 2+	1004.513	1.0	–
C20 2+	1073.0431	1.5	1.2
C20 3+	715.6971	0.4	
C21 2+	1137.0919	2.6	2.7
C21 3+	758.3955	0.5	
C23 3+	833.4448	0.6	1.2
C25 3+	884.8025	0.1	–
C26 3+	903.8102	0.7	0.8
Z7	929.4108	0.9	0.9
Z12 2+	745.3626	0.5	–
Z13 2+	809.392	0.6	–
Z15 2+	930.4556	0.9	–
Z16 2+	994.5033	1.0	–
Z19 2+	1179.6148	1.4	1.5
Z20 3+	829.4312	0.6	2.2
Z21 3+	878.4538	0.3	0.4
Z28 3+	1137.9461	2.3	2.3

The RMS errors are 1.2 and 1.8 ppm, respectively.

– = Not observed upon deconvolution.

It is readily evident that the combined fragmentation information allows for extensive sequence characterization despite very modest spectral averaging. ETD reasonably covers the N- and C-terminal regions of the protein whereas more coverage of the center is achieved by CID, demonstrating the utility of combining fragmentation techniques as well as their complementarity. In the case of CID of the non-variant protein (Figure 3c and d), the data were sufficient to localize the non-variant amino acid Thr160 to the protein sequence. In the CID spectrum of the variant protein, position 160 is flanked by fragments b_151_ (observed in both the 21+ and 22+ charge states) and b_172_^24+^. Because this region contains only one candidate Thr residue (and no Met residues, as a Met→Tyr substitution differs only slightly in mass shift), we can infer that this is the site of the Thr→Arg substitution. The verification of the amino acid sequences of the proteins by experimental MS and MS/MS data is an important step prior to X-Ray crystallography investigations.

### Crystallographic Analysis

**Figure 5:**
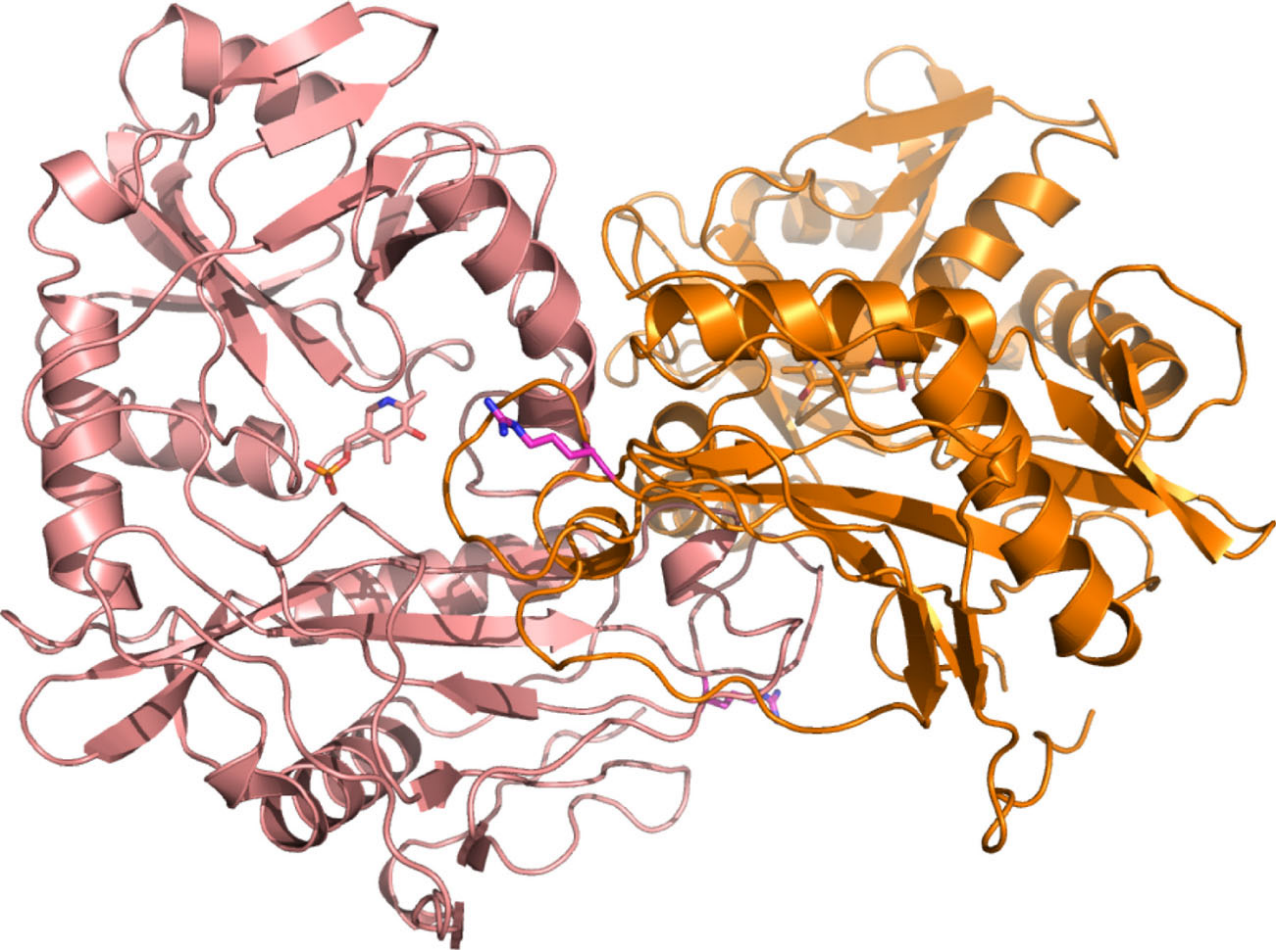
The T186R variant structure (T159R for numbering in the PDB file). The two monomers of the T186R mutant are colored in coral and orange, respectively. Compared with the non-variant BCAT2, it crystallizes in a different space group at exactly the same condition (pH 6.5)

**Figure 6:**
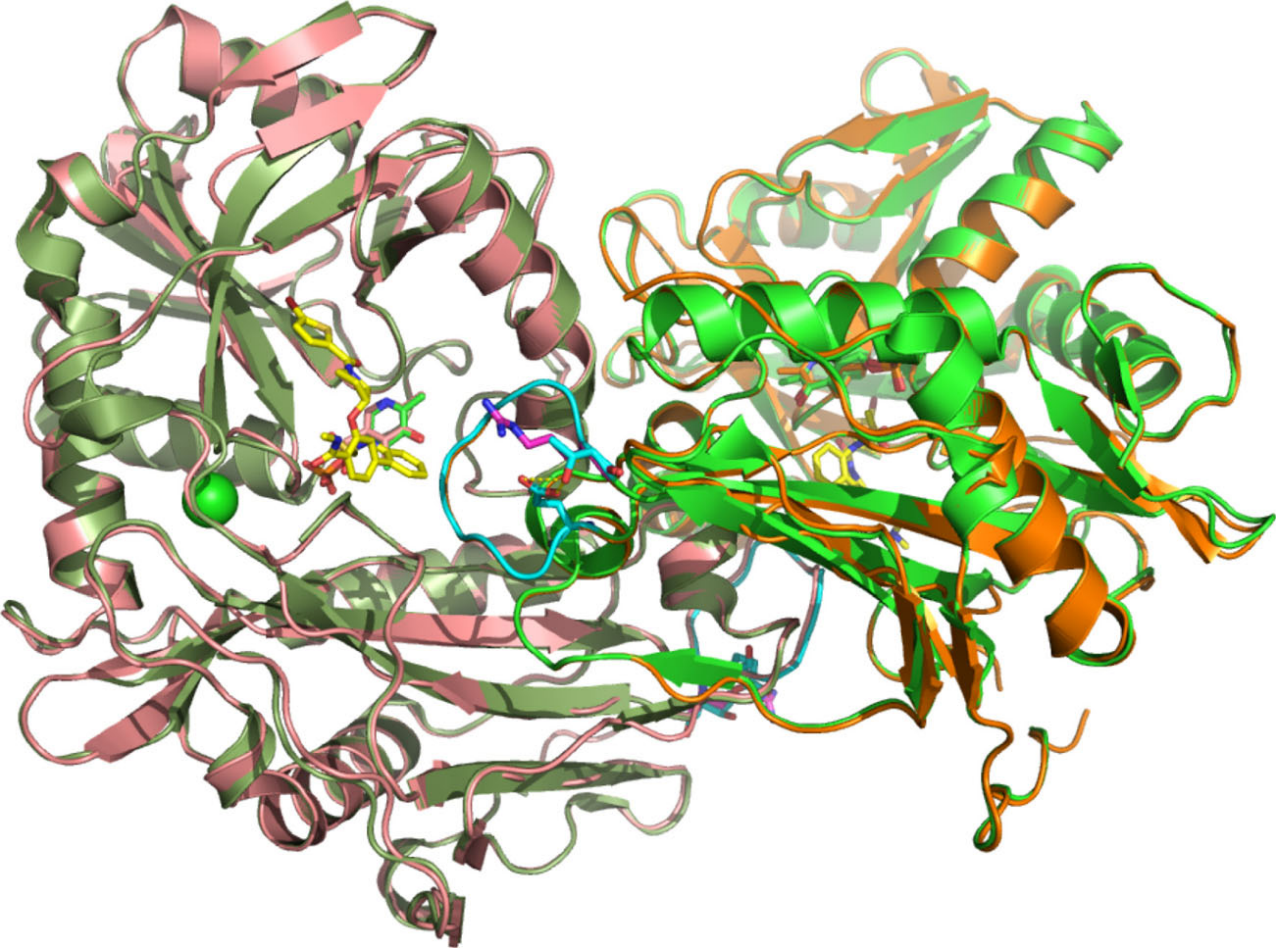
The T186R variant structure (T159R for numbering in the PDB file) overlaid on the BCAT2 inhibitor bound structure (PDB code 5CR5) described in [37]. The two different monomers of the T186R mutant are colored in coral and orange, respectively. The 5CR5 structure is colored in shades of green. The T186 loop is colored in cyan with R186 in magenta and the inhibitor in yellow

There are several BCAT2 structures determined, each with two molecules found in an asymmetric unit forming a biological assembly [37, 45]. Here, we present the first BCAT2 T186R variant structure with a novel trigonal crystal lattice, with only one molecule in the asymmetric unit (Figure 5). The biological assembly, the dimer, is formed over a 2-fold crystallographic symmetry axis. The T186R amino acid substitution is found close to the interface of the dimer. No structural difference is observed at position 186. The CB and CG2 (methyl) carbons of threonine 186 are almost perfectly overlapping with the same carbons in the arginine side chain in the BCAT2 T186R structure. Superposition of the BCAT2 T186R protein structure with the WT BCAT2 monomer (PDB code 5CR5, resolution 1.61 Å) yields a RMSD of 0.268 Å (comparing 334 CA atoms in each protein structure); thus, the overall protein conformation of the BCAT2 monomers are very similar. The crystallographic experiments confirm the hypothesis generated by in silico structural homology modeling as detailed in Lichti et al. [3]. Because the biological unit is a dimer, the dimer needs to be analyzed. Less than 6 Å away from the T186R mutant, the side chain of K59 from the symmetry-related BCAT2 monomer is found. One can speculate that repulsion of these amino acid side chains may destabilize the BCAT2 T186R dimer. Destabilization of the BCAT2 dimer may affect the enzymatic activity of the BCAT2 variant depending on the pH of the environment, yielding altered enzyme kinetics. This is of relevance to the development of a precision drug for the BCAT2 variant because hypoxic niches and lowered pH are frequently associated with GBM tumors (Figure 6).

## Conclusions

The further development of our therapeutic targets requires structural studies of intact protein variants by high resolution mass spectrometry, X-Ray crystallography, and structural modeling to provide physical coordinates for fragment-based drug design or therapeutic antibody development. Recent improvements in FT-ICR MS instrumentation, in speed of analysis, sensitivity, resolution, and extension of the upper mass range of ions that can be measured are a strong driver of protein characterization today. When these characteristics are combined with the complementary nature of MS/MS techniques that have been developed in the past decade by Håkansson et al. [8–12, 46] as well as other investigative consortia, high field, high sensitivity FT-ICR MS(/MS) is a formidable analytical technology. Synergistic data were obtained by FT-ICR MS/MS and X-Ray crystallographic studies, providing both confirmation of the sequences of the expressed proteins and demonstrating differences in crystallization patterns due to a single amino acid substitution. Taken together, our results confirm our hypothesis that the T186R variant has an altered kinetic profile compared with the non-variant. We expect that these improved technologies will expedite the development of targets yet to be identified in proteomic and proteogenomic screens. In the case of the BCAT2 T186R variant protein, comparative metabolomics screens are indicated to determine how the production of glutamate, a neurotoxic metabolite, may be affected by biochemical conditions relevant to GBM. Other technologies that will benefit the pursuit of precision medicine are native MS of proteins and protein–drug complexes and fragment-based drug design by use of MS as well as bright light sources, such as selected beamlines at MAX IV Laboratory.

## Electronic supplementary material

Below is the link to the electronic supplementary material.

Supplementary Table 1 Statistics for Data Collection and Structural Determination of BCAT2 Proteins (DOCX 19 kb)
