# Supplementary material for: Intact Protein Analysis at 21 Tesla and X-Ray Crystallography Define Structural Differences in Single Amino Acid Variants of Human Mitochondrial Branched-Chain Amino Acid Aminotransferase 2 (BCAT2)
Source: J Am Soc Mass Spectrom. 2017 Jul 5;28(9):1796–804. doi: 10.1007/s13361-017-1705-0 (PMC5556139; doi:10.1007/s13361-017-1705-0)
Supplement: Supplementary file 1 — Statistics for Data Collection and Structural Determination of BCAT2 Proteins (DOCX 19 kb) [file 13361_2017_1705_MOESM1_ESM.docx]

**Appendices (Supporting Information)**

**S Table 1. Statistics for Data Collection and Structural Determination of BCAT2 Proteins**
